# Complete Genome Sequence of the Probiotic *Lactobacillus plantarum* Strain ZJ316

**DOI:** 10.1128/genomeA.00094-13

**Published:** 2013-03-21

**Authors:** Xuan Li, Qing Gu, Xiuyu Lou, Xiaomei Zhang, Dafeng Song, Lei Shen, Yizhen Zhao

**Affiliations:** Key Laboratory for Food Microbial Technology of Zhejiang Province, College of Food Science and Biotechnology, Zhejiang Gongshang University, Hangzhou, China

## Abstract

*Lactobacillus plantarum* strain ZJ316, a probiotic strain with several functions, was isolated from healthy newborn infant fecal samples. Here we report the finished and annotated genome sequence of this organism.

## GENOME ANNOUNCEMENT

*Lactobacillus plantarum* is a versatile facultative heterofermentative lactic acid bacterium (LAB) that is encountered in a variety of environmental niches (1–3) and commonly used as a probiotic. *L. plantarum* strain ZJ316, originally isolated from healthy infant fecal samples, has many probiotic properties, such as significant improvement of pig growth and pork quality and antimicrobial activity against various pathogens *in vitro*, such as *Micrococcus luteus*, *Bacillus subtilis*, *Staphylococcus aureus*, *Escherichia coli*, *Salmonella enterica*, *Listeria monocytogenes*, and others (4). Here, we report the genome sequence of *L. plantarum* strain ZJ316.

Whole-genome sequencing of *L. plantarum* strain ZJ316 was performed (BGI-Shenzhen, Shenzhen, China) with a shotgun strategy. A total of 857 Mb of next-generation Illumina paired-end 90-bp reads was generated by sequencing genome shotgun libraries of different fragment lengths (500 bp, 2 kb, and 6 kb) that covered 267.5-fold of the genome. The assembly, performed by SOAPdenovo (5, 6), consisted of 239 contigs and 61 scaffolds and accounted for approximately 99.99% of the estimated *L. plantarum* strain ZJ316 genome. Contigs were further assembled using paired-end reads together with mapping to the reference genome of *L. plantarum* strain WCFS1 (1, 7), resulting in the assembled draft genome with a superscaffold and 2 contigs. Physical gaps were filled through sequencing of PCR products that spanned these regions. To evaluate the single-base accuracy of the assembled genome sequence, we realigned all the usable sequencing reads onto the scaffolds using SOAPaligner/soap2 (8).

*L. plantarum* strain ZJ316 has one circular chromosome of 3,203,964 bp, with a G+C content of 44.65%, and three plasmids, pLP-ZJ101 (15,167 bp with 40.18% G+C), pLP-ZJ102 (39,116 bp with 38.69% G+C), and pLP-ZJ103 (41,508 bp with 39.50% G+C). There are 3,159 protein-coding genes, 15 rRNA operons, and 61 tRNAs in the chromosome and 117 coding genes in the plasmid.

Compared with the published genomes of *L. plantarum* strains WCFS1 (1, 7), ATCC 14917 (GenBank accession number NZ_ACGZ00000000.2), JDM1 (9), ST-III (10), and NC8 (11), most of the orthologous genes of ZJ316 have >93% nucleotide sequence identity. Some prophage sequences and locations of ZJ316 are novel. There are more than 200 unique genes that are present in ZJ316. These include RNA-directed DNA polymerases and clustered regularly interspaced short palindromic repeat (CRISPR)-associated protein genes, which encode enzymes involved in the DNA repair pathway. There are also new candidates, which contain the genes for glucarate dehydratase, tagatose 1,6-diphosphate aldolase 1, gluconate transport protein, the phosphotransferase system (PTS) family cellobiose porter, and other carbohydrated-related proteins, involved in carbohydrate metabolism.

Many sugar metabolism and transport genes in WCFS1 and other *L. plantarum* strains were lost in ZJ316, such as gene clusters for biosynthesis and transport of arabinose, rhamnose, mannose, and other carbohydrates.

In conclusion, the genes specific to *L. plantarum* strain ZJ316 may reflect niche differences between *L. plantarum* strain ZJ316 and the other *L. plantarum* strains, suggesting that *L. plantarum* strain ZJ316 may have different carbohydrate utilization to adapt to the human gastrointestinal tract. The genome sequence gives us a basis to further elucidate the functional mechanisms of its probiotic properties.

### Nucleotide sequence accession number. 

The complete nucleotide sequence of the *Lactobacillus plantarum* ZJ316 chromosome has been deposited in GenBank under the accession number CP004082.
